# Glucose and triglyceride excursions following a standardized meal in individuals with diabetes: ELSA-Brasil study

**DOI:** 10.1186/s12933-015-0181-8

**Published:** 2015-02-13

**Authors:** Bárbara P Riboldi, Vivian C Luft, Cristina D de Castilhos, Letícia O de Cardoso, Maria I Schmidt, Sandhi M Barreto, Maria F de Sander, Sheila M Alvim, Bruce B Duncan

**Affiliations:** Graduate Studies Program in Epidemiology, School of Medicine, Federal University of Rio Grande do Sul, Porto Alegre, RS Brazil; Food and Nutrition Research Center, Hospital de Clínicas de Porto Alegre, Federal University of Rio Grande do Sul, Rua Ramiro Barcelos 2600/419, Porto Alegre, RS 90035-003 Brazil; National School of Public Health, Oswaldo Cruz Foundation, Rio de Janeiro, RJ Brazil; Graduate Studies Program in Public Health, Faculty of Medicine, Federal University of Minas Gerais, Belo Horizonte, MG Brazil; Internal Medicine Department, Faculty of Medicine, Federal University of Minas Gerais, Belo Horizonte, MG Brazil; Institute of Collective Health, Universidade Federal da Bahia, Salvador, Brazil

## Abstract

**Objective:**

To assess glucose and triglyceride excursions 2 hours after the ingestion of a standardized meal and their associations with clinical characteristics and cardiovascular complications in individuals with diabetes.

**Research design and methods:**

Blood samples of 898 subjects with diabetes were collected at fasting and 2 hours after a meal containing 455 kcal, 14 g of saturated fat and 47 g of carbohydrates. Self-reported morbidity, socio-demographic characteristics and clinical measures were obtained by interview and exams performed at the baseline visit of the ELSA-Brasil cohort study.

**Results:**

Median (interquartile range, IQR) for fasting glucose was 150.5 (123–198) mg/dL and for fasting triglycerides 140 (103–199) mg/dL. The median excursion for glucose was 45 (15–76) mg/dL and for triglycerides 26 (11–45) mg/dL. In multiple linear regression, a greater glucose excursion was associated with higher glycated hemoglobin (10.7, 95% CI 9.1**–**12.3 mg/dL), duration of diabetes (4.5; 2.6**–**6.4 mg/dL, per 5 year increase), insulin use (44.4; 31.7**–**57.1 mg/dL), and age (6.1; 2.5**–**9.6 mg/dL, per 10 year increase); and with lower body mass index (−5.6; −8.4**–** -2.8 mg/dL, per 5 kg/m^2^ increase). In adjusted logistic regression models, a greater glucose excursion was marginally associated with the presence of cardiovascular comorbidities (coronary heart disease, myocardial infarction and angina) in those with obesity.

**Conclusions:**

A greater postprandial glycemic response to a small meal was positively associated with indicators of a decreased capacity for insulin secretion and negatively associated with obesity. No pattern of response was observed with a greater postprandial triglyceride excursion.

## Introduction

Diabetes prevalence is increasing worldwide, mainly due to the aging of the population and the epidemic rise in obesity [1], the two main determinants of type 2 diabetes. Diets containing high levels of animal fat and a high glycemic index are associated with both diabetes mellitus (DM) and cardiovascular disease (CVD), a major complication of diabetes [2-4]. Hyperglycemia is a major risk factor for the development of chronic microvascular (retinopathy and nephropathy) and macrovascular (ischemic cardiomyopathy, stroke, and peripheral vascular disease) complications [5]. Postprandial hyperglycemia contributes importantly to overall hyperglycemia and cardiovascular complications [6].

A 2 h post-load plasma glucose during a 75-g OGTT is more predictive of overall mortality than fasting glucose [7]. Postprandial hyperglycemia, measured after breakfast, lunch and dinner, is an independent risk factor for CVD [8] and mortality [9] among individuals with diabetes. Additionally, postprandial hypertriglyceridemia has been shown to be a risk factor for CVD among individuals without diabetes [10] and is associated with an increased intima-media thickness of the carotid artery in individuals with diabetes [11]. However, few large studies have evaluated glucose and triglyceride post-meal excursions in individuals with diabetes, and their association with diabetes comorbidities.

This study aims to assess glucose and triglyceride excursions 2 hours after the ingestion of a standardized meal and their associations with clinical characteristics and the presence of cardiovascular complications in individuals with diabetes.

## Research design and methods

This is a cross-sectional analysis of 2008–2010 baseline data from the Brazilian Longitudinal Study of Adult Health (ELSA-Brasil), a multicenter cohort study of 15,105 public sector employees (active or retired) in six cities in three different regions of Brazil [12]. Of these, 1496 reported a previous diagnosis of diabetes, and 958 (64%) performed the meal test. We excluded 46 participants because of incomplete meal ingestion or missing values for fasting or post-meal plasma glucose and triglycerides, and 14 participants reporting diabetes onset before the age of 40 with insulin being their first medication, characteristics suggesting type I diabetes. Thus, the final analysis was performed on 898 diabetic subjects. ELSA-Brasil was approved by the respective Ethics and Research Committee of each participating institution, and participants gave written consent prior to entering the study.

Sociodemographic data, a past medical history of diabetes and other diseases, and drug use were obtained through a standardized interview. Anthropometric data – height, weight, waist and hip circumferences and blood pressure – were measured, blood pressure being obtained three times with the mean of the last two measurements used in analyses.

A 12 hour fasting blood sample was drawn by venipuncture in the morning period following standardized procedures for samples collection and processing [13]. Participants were instructed not to use medicines on the morning of examinations until after this collection. The standardized meal was administered shortly thereafter, and an additional sample was drawn 2 hours later. The meal consisted of four slices (30 grams) of industrialized toast (Bauducco®), four cubes (20 grams) of processed cheese (Polenghinho®), and a box (200 ml) of an orange-flavored drink (Kapo®), totaling, according to labels, 454.8 kcal, and containing 24.4 grams of lipids (13.8 grams of saturated fat), 46.8 grams of carbohydrates, 12.0 grams of protein, and 570 mg of sodium.

Blood specimens were processed locally and then sent to the central ELSA-Brasil laboratory for analysis. Glucose was determined by the hexokinase enzymatic method. Total cholesterol, triglycerides, HDL-cholesterol and LDL-cholesterol were estimated by the enzymatic colorimetric method. Insulin was determined by chemiluminescence (sandwich immunoassay) and C-reactive protein by an automated immunochemical method [14]. Glucose and triglyceride post-load excursions were defined as the difference between the corresponding 2 h post-load and fasting values.

Raised blood pressure was defined as systolic blood pressure ≥ 130 mmHg, a diastolic blood pressure ≥ 85 mmHg [15], or confirmed use of antihypertensive medication [16]; postural hypotension as a decrease of ≥ 20 mmHg for systolic or ≥ 10 mmHg for diastolic blood pressure when changing from supine to standing position [17]. Coronary disease was defined as a history of angina, myocardial infarction, angioplasty or bypass surgery; any cardiovascular complication as coronary disease, heart failure, or stroke. Abdominal obesity was considered present when waist circumference was > 102 cm for men and > 88 cm for women; hypertriglyceridemia when triglycerides > 150 mg/dL; and low HDL-cholesterol when HDL-cholesterol was < 40 mg/dL for men and < 50 mg/dL for women [16]. Continuous variables were expressed as mean (standard deviation) or median (interquartile range), according to their distribution, with Spearman correlation coefficients being used to assess associations. ANCOVA was used for comparison of glucose and triglyceride excursions between groups with different demographic and clinical characteristics.

Linear regression models were constructed to investigate associations of clinical characteristics with glucose and triglyceride excursions, linearity being assessed using the Box-Tidwell test. Logistic regression models were fitted to investigate associations of glucose and triglyceride excursions with the presence of chronic diseases. Multicollinearity among variables was assessed by the variance inflation factor.

A significance level of 5% was adopted. Analyses were performed using SAS software (Statistical Analysis System, SAS Institute Inc., Cary, NC), version 9.3.

## Results

Table 1 presents clinical and laboratory characteristics of the 898 participants with diabetes. Of note, median age was 59.5 (54.0 to 65.0) years, and most were overweight (42.7%) or obese (41.8%). Median duration of diabetes was 6 (3.0-12.0) years, and 15.6% were using insulin and 76.6% other medications (principally metformin and sulfonylureas) for diabetes. The median for fasting glucose was 150.5 (123.0 to 198.0) mg/dL and for post-load glucose 200.0 (146.0 to 274.0) mg/dL. The median glucose excursion was 44.0 mg/dL, with interquartile range from 15.0 to 76.0 mg/dL. The median for fasting plasma triglycerides was 140 (103.0 to 199.0) mg/dL and for post-load triglycerides 172.0 (128.0 to 237.0) mg/dL. The median triglyceride excursion was 26.0 mg/dL, with interquartile range from 11.0 to 45.0 mg/dL. Only 16.5% of participants reported a cardiovascular comorbidity.

**Table 1:** **Characteristics of participants with self-reported diabetes who consumed a test meal (n = 898)**

**Characteristic**	**Median or N**	**Interquartile range or %**
Age (years)	59.5	54.0-65.0
Race		
Black	220	25.1%
Brown (“pardos”)	230	26.2%
White	383	43.7%
Body mass index (kg/m^2^)	28.9	26.1-32.4
Waist circumference (cm)		
Men	100.6	93.7-107.9
Women	96.9	89.8-106.8
Systolic blood pressure (mmHg)	128.3	117.0-141.5
Diastolic blood pressure (mmHg)	78.0	71.0-85.0
Raised blood pressure	699	78.2%
Duration of diabetes (years)	6	3.0-12.0
Self-referred insulin treatment	131	15.6%
Self-referred oral hypoglycemic treatment alone	619	76.6%
Self-referred statin treatment	285	31.7%
Self-referred use of other lipid-lowering drugs	33	3.7%
Glycated hemoglobin (%)	6.6	5.8-7.9
Fasting glucose (mg/dL)	150.0	123.0-198.0
2 h post-load glucose (mg/dL)	200.0	146.0-274.0
Glucose excursion (mg/dL)^†^	44.0	15.0-76.0
Fasting triglycerides (mg/dL)	140.0	103.0-199.0
2 h post-load triglycerides (mg/dL)	172.0	128.0-237.0
Triglyceride excursion (mg/dL)^†^	26.0	11.0-45.0
Fasting insulin (μU/mL)^‡^	8.8	5.1-13.9
Cholesterol (mg/dL)	199.0	173.0-231.0
HDL-cholesterol (mg/dL)	49.0	42.0-59.0
LDL-cholesterol (mg/dL)	116.0	94.0-142.0
Postural hypotension	23	2.6%
Self-reported		
Stroke	22	2.5%
Heart failure	43	4.8%
Angina	66	7.4%
Myocardial infarction	51	5.7%
Coronary disease	108	12.0%
Any cardiovascular complication	148	16.5%

ELSA-Brasil, 2008–2010.

Small variations in the total number of individuals are due to missing data.

^†^2 h post-load level minus fasting level.

^‡^Only among those not on insulin use (n = 683).

The strongest correlations with the post-load glucose excursion were seen with fasting glucose (r = 0.43, p <0.01), post-load glucose (r = 0.83 p <0.01), and glycated hemoglobin (r = 0.53, p <0.01). Lesser correlations were observed with duration of diabetes (r = 0.34, p <0.01), waist-hip ratio (r = 0.16, p <0.01), systolic blood pressure (r = 0.17 p <0.01) and age (r = 0.11, p <0.01). Correlations for triglyceride excursion were generally weaker, with no correlation being observed with fasting triglycerides and a moderate correlation with post-load triglycerides (r = 0.34, p <0.01). Weak correlations were present with fasting glucose (r = 0.11, p <0.01), post-load glucose (r = 0.09, p <0.01), glycated hemoglobin (r = 0.12, p <0.01) and fasting insulin (r = 0.09, p <0.01), as illustrated in Table 2.

**Table 2:** **Spearman correlation coefficients between clinical and laboratory variables in patients with diabetes during a test meal (n = 898)**

	**Fasting glucose**	**Post-load glucose**	**Glucose excursion** ^**†**^	**Fasting triglycerides**	**Post-load triglycerides**	**Triglyceride excursion** ^**†**^
Fasting glucose	---	0.83**	0.43**	0.26**	0.29**	0.11**
Post-load Glucose	0.83**	---	0.83**	0.19**	0.22**	0.09**
Glucose excursion	0.43**	0.83**	---	0.05	0.08*	0.04
Fasting triglycerides	0.26**	0.19**	0.05	---	0.94**	0.06
Post-load Triglycerides	0.29**	0.22**	0.08	0.94**	---	0.34**
Triglyceride excursion	0.11**	0.09**	0.04	0.06	0.34**	---
Glycated hemoglobin	0.73**	0.76**	0.53**	0.15**	0.20**	0.12**
Fasting insulin	−0.07*	−0.01	0.05	0.20**	0.21**	0.09**
C-reative Protein	0.13**	0.12**	0.06	0.11**	0.11**	0.05
Age	−0.08*	−0.01	0.11**	−0.09*	−0.10*	−0.05
Body mass index	0.04	−0.01	−0.06	0.20**	0.18**	0.04
Waist circumference	0.11**	0.07*	−0.01	0.22**	0.20**	0.01
Waist to hip ratio	0.20**	0.21**	0.16**	0.25**	0.23**	−0.01
Duration of Diabetes	0.16**	0.31**	0.34**	−0.05	−0.06	−0.06
Systolic blood pressure	0.22**	0.22**	0.17**	0.09**	0.09**	0.02
Diastolic blood pressure	0.24**	0.17**	0.07*	0.11**	0.12**	0.04

ELSA-Brasil, 2008–2010.

^†^Two-hour post-load level minus fasting level; *P < 0.05; **P < 0.01.

In crude analyses (Table 3), the magnitude of the glucose excursions varied with several clinical characteristics. Higher glucose excursions were found among those with HbA1_C_ values greater than 7%, use of insulin, postural hypotension, and self-reported cardiovascular comorbidity. Lower excursions were found among those with abdominal obesity and also those receiving only oral hypoglycemic treatment. No statistically significant associations were seen with triglyceride excursions.

**Table 3:** **Difference between glucose and triglycerides excursions in individuals with diabetes according to clinical and laboratory characteristics (n = 898)**

	**Glucose excursion (mg/dL)**	**95% CI**	**P**	**Triglycerides excursion (mg/dL)**	**95% CI**	**P**
Obesity (BMI ≥ 30 kg/m^2^)						
Yes	49.2	44.3; 54.2	0.26	27.8	23.7; 31.8	0.74
No	52.2	48.0; 56.4		26.9	23.4; 30.3	
Abdominal obesity						
Yes	48.8	44.8; 52.9	0.10	28.3	25.0; 31.7	0.31
No	54.3	49.2; 59.4		25.8	21.4; 29.8	
A1c > 7%						
Yes	75.9	71.5; 80.4	<0.001	27.5	23.5; 31.6	0.86
No	33.2	29.4; 37.0		27.1	23.6; 30.5	
Oral hypoglycemic treatment (without insulin)						
Yes	46.1	42.3; 49.8	<0.001	27.9	24.7; 31.1	0.17
No	75.5	68.8; 82.3		23.3	17.5; 29.1	
Insulin use						
Yes	96.7	88.9; 104.4	<0.001	21.2	14.3; 28.2	0.09
No	44.6	41.3; 48.0		27.9	24.9; 30.9	
Postural hypotension						
Yes	82.0	62.1; 101.8	0.001	29.8	13.1; 46.4	0.77
No	49.8	46.5; 53.0		27.3	24.6; 30.0	
Any cardiovascular complication						
Yes	63.7	55.9; 71.5	0.001	27.0	20.6; 33.4	0.94
No	48.4	45.0; 51.2		27.3	24.5; 30.2	
Myocardial infarction						
Yes	72.7	59.4; 86.0	0.001	29.9	19.1; 40.8	0.62
No	49.7	46.4; 52.9		27.1	24.4; 29.8	
Angina						
Yes	61.3	49.5; 73.0	0.07	27.6	17.9; 37.2	0.96
No	50.0	46.8; 53.4		27.3	24.6; 30.0	
Heart failure						
Yes	65.7	51.1; 80.2	0.04	28.6	16.8; 40.4	0.82
No	50.2	47.0; 53.5		27.2	24.5; 29.9	
Coronary disease						
Yes	63.5	54.4; 72.7	0.004	27.6	20.1; 35.2	0.91
No	49.2	45.9; 52.6		27.2	24.4; 30.0	
Stroke						
Yes	74.6	54.3; 94.9	0.02	23.9	7.0; 40.7	0.69
No	50.4	47.1; 53.6		27.3	24.7; 30.0	
Raised blood pressure						
Yes	51.9	48.3; 55.5	0.32	26.9	23.9; 29.9	0.73
No	50.3	41.2; 54.8		28.0	22.4; 33.7	
Hypertriglyceridemia						
Yes	50.6	45.9; 55.3	0.84	27.2	23.3; 31.1	0.98
No	51.3	46.9; 55.6		27.3	23.7; 30.8	
Low HDL-C						
Yes	49.0	43.2; 54.9	0.44	28.9	24.1; 33.6	0.43
No	51.8	48.0; 55.6		26.5	23.4; 29.7	

ELSA-Brasil, 2008–2010.

Table 4 shows analyses of glucose and triglyceride excursions with adjustment for covariates. In the more adjusted analysis, glucose excursion was 7.6 mg/dL greater in whites, 6.1 mg/dL greater for each 10 years increase in age; 4.5 mg/dL greater for each five year increase in duration of diabetes; 10.7 mg/dL greater for each 1% increase of glycated hemoglobin; and 44 mg/dL greater in the presence of insulin use. Glucose excursion decreased by 5.6 mg/dL for each 5 kg/m^2^ increase in body mass index. For triglyceride excursions, as in the crude analyses, no variation in magnitude was observed according to the factors analyzed.

**Table 4:** **Adjusted** ^**†**^ **differences between glucose and triglyceride excursions in individuals with diabetes (n = 898)**

**Glucose excursion**	**Model 1**			**Model 2**		
	**mg/dL**	**95% CI**	**p**	**mg/dL**	**95% CI**	**p**
Race *(white vs. non-white)*	5.3	−0.5; 11.1	0.07	7.6	1.9; 13.3	0.01
Sex *(male vs. female)*	−5.2	−10.9; 0.5	0.07	−2.1	−7.8; 3.5	0.45
Age *(10 years)*	8.4	4.9; 11.8	<0.001	6.1	2.5; 9.6	<0.001
Glycated hemoglobin *(%)*	13.3	11.7; 14.9	<0.001	10.7	9.1; 12.3	<0.001
Body mass index *(5 kg/m* ^*2*^ *)*				−5.6	−8.4; −2.8	<0.001
Duration of diabetes *(5 years)*				4.5	2.6; 6.4	<0.001
Other medication for diabetes *(yes vs. no)*				9.4	−1.2; 20.1	0.08
Insulin use *(yes vs. no)*				44.4	31.7; 57.1	<0.001
**Triglyceride excursion**	**Model 1**			**Model 2**		
	**mg/dL**	**95% CI**	**p**	**mg/dL**	**95% CI**	**p**
Race *(white vs.non-white)*	3.3	−2.2; 8.8	0.24	3.7	−2.1; 9.6	0.21
Sex *(male vs. female)*	2.1	−3.3; 7.5	0.45	3.0	−2.9; 8.7	0.33
Age *(10 years)*	−2.8	−6.1; 0.4	0.09	−3.3	−7,0; 0.3	0.07
Glycated hemoglobin *(%)*	0.87	−0.8; 2.2	0.33	0.6	−1.1; 2.3	0.48
Body mass index *(5 kg/m* ^*2*^ *)*				1.0	−1.9; 3.9	0.51
Duration of diabetes *(5 years)*				0.2	−1.8; 2.3	0.82
Other medication for diabetes *(yes vs. no)*				−1.3	−12.3; 9.8	0.82
Insulin use *(yes vs. no)*				−8.1	−21.3; 5.0	0.22

ELSA-Brasil, 2008–2010.

^†^Through linear regression model for other variables shown in each model in the table.

Table 5 presents the associations of glucose and triglyceride excursions with cardiovascular comorbidities, when adjusted through logistic regression. The results are presented both globally and with participants stratified into obese and non-obese categories, and odds ratios are expressed for a change of 25 mg/dL in glucose and triglyceride excursions. Covariate control was limited given the small number of outcomes observed. In this regard, only age-adjusted models are presented for the more specific outcomes (myocardial infarction, angina, stroke, and heart failure). Associations for the total sample for both glucose and triglyceride excursions were minimal. However, when the associations were investigated in subgroups, a relatively consistent pattern of small though generally not statistically significant associations was frequently seen for obese individuals. With the exception of stroke, the outcome with fewest cases, all associations were stronger among the obese, the excursion – obesity interaction being statistically significant for the triglyceride excursion for many of the outcomes.

**Table 5:** **Adjusted** ^**†**^ **associations of glucose and triglyceride excursions (of 25 mg/dL) with clinical outcomes (n = 898)**

**Glucose excursion**	**Model**	**Global**			**Obese (n=375)**			**Non-obese (n=523)**			**Interaction** ^**‡**^
		**OR**	**95% CI**	**p**	**OR**	**95% CI**	**p**	**OR**	**95% CI**	**p**	**p**
Any cardiovascular complication	1	1.15	(1.06-1.25)	<0.01	1.19	(1.01-1.39)	0.04	1.15	(1.03-1.27)	0.01	0.86
	2	1.06	(0.95-1.17)	0.32	1.12	(0.92-1.36)	0.26	1.03	(0.89-1.19)	0.94	0.35
Heart disease	1	1.13	(1.03-1.25)	0.01	1.22	(1.03-1.46)	0.02	1.10	(0.98-1.25)	0.11	0.38
	2	1.04	(0.91-1.18)	0.57	1.20	(0.96-1.50)	0.12	0.96	(0.82-1.14)	0.65	0.11
Myocardial infarction	1	1.21	(1.06-1.38)	<0.01	1.32	(1.03-1.68)	0.03	1.17	(1.00-1.36)	0.05	0.42
Angina	1	1.11	(0.99-1.25)	0.09	1.21	(0.99-1.48)	0.06	1.07	(0.92-1.25)	0.41	0.36
Stroke	1	1.23	(1.02-1.48)	0.03	1.08	(0.63-1.84)	0.78	1.25	(1.-1.47)	0.05	0.50
Heart failure	**1**	1.15	(1.00-1.33)	0.06	1.19	(0.93-1.52)	0.16	1.15	(0.95-1.38)	0.15	0.90
**Triglyceride excursion**		**Global**			**Obese (n=375)**			**Non-obese (n=523)**			**Interaction** ^**‡**^
		**OR**	**95% CI**	**p**	**OR**	**95% CI**	**p**	**OR**	**95% CI**	**p**	**p**
Any cardiovascular complication	1	1.00	(0.90-1.12)	0.94	1.11	(0.91-1.35)	0.32	0.93	(0.81-1.08)	0.34	0.15
	2	1.01	(0.90-1.13)	0.87	1.15	(0.93-1.43)	0.20	0.91	(0.78-1.06)	0.21	0.06
Heart disease	1	1.02	(0.89-1.16)	0.80	1.23	(0.96-1.58)	0.10	0.91	(0.78-1.07)	0.24	0.04
	2	1.02	(0.90-1.17)	0.72	1.25	(0.95-1.63)	0.11	0.91	(0.77-1.07)	0.24	0.04
Myocardial infarction	1	1.07	(0.87-1.32)	0.52	1.12	(0.79-1.59)	0.53	1.05	(0.80-1.36)	0.73	0.79
Angina	1	1.01	(0.85-1.19)	0.93	1.36	(1.01-1.83)	0.04	0.86	(0.72-1.02)	0.09	0.01
Stroke	1	0.96	(0.76-1.21)	0.75	0.89	(0.64-1.24)	0.49	0.98	(0.72-1.33)	0.92	0.76
Heart failure	1	1.03	(0.84-1.27)	0.76	1.09	(0.79-1.49)	0.60	0.97	(0.75-1.26)	0.84	0.56

ELSA-Brasil, 2008–2010.

^†^Through logistic regression, as follows:

Model 1: age.

Model 2: model 1 + sex, glycated hemoglobin, duration of diabetes, use of insulin.

^‡^Excursion versus obesity interaction.

In multiply-adjusted analyses in the obese strata, the presence of any cardiovascular comorbidity was 12% (−8% to 36%) more frequent for every 25 mg/dL greater glucose excursion, and 15% (−7% to 43%) more frequent for every 25 mg/dL greater triglyceride excursion. In this strata, an increase in the glucose excursion of this magnitude was associated with a 32% increase in the age-adjusted risk of having had a myocardial infarction (OR = 1.32, 95% CI 01.03 to 1.68), and an increase in the triglyceride excursion of this magnitude with a 36% increase in the age-adjusted risk of having a history of angina (OR = 1.36, 95% CI 1.01 to 1.83).

## Discussion

The metabolic response to a test meal of approximately 450 kcal, roughly equivalent to a breakfast meal, led to 2 h glucose and triglyceride values 30% and 20% above those of fasting. However, the excursions varied widely, with those in the 75th percentile having an excursion approximately four times that of those in the 25th percentile for both glucose and triglycerides. Clinical correlates of a greater glucose excursion suggest that it reflects a decreased capacity for insulin secretion, whereas no correlates of the triglyceride excursion were identified. In overall analyses, no associations were found between a greater excursion and a history of cardiovascular comorbidity. However, the relative risk of presenting a comorbidity with a larger excursion was generally greater in the obese, though this risk was never of a large magnitude.

The major predictors of greater glycemic excursion – greater age, duration of diabetes, glycated hemoglobin and, especially, the use of insulin – suggest that the main determinant of the post meal blood glucose excursion was a relative insulin deficiency. That obesity associated with a lesser excursion perhaps similarly reflects greater preservation of the capacity for insulin secretion. Individuals with a greater excursion are presumably further along the progressive course of beta-cell dysfunction which characterizes the natural history of type 2 diabetes [18]. Bonora et al. [19] when evaluating cross-sectional associations of blood glucose two hours after breakfast, lunch, and dinner, measured at home in 3,284 diabetic subjects, found similarly that age and diabetes duration were associated with greater excursions, and obesity with lesser ones.

On the other hand, the larger associations with clinical outcomes found in obese individuals suggest that the clinical impact of glycemic and triglyceride excursions may be greater for these individuals. This finding is consistent with the previous finding of a greater association of rapidly absorbed carbohydrate in the diet with circulating levels of C-reactive protein [20].

Few previous studies have evaluated the impact of postprandial blood glucose excursions on cardiovascular outcomes. Cavalot et al. [8] followed 529 individuals with diabetes for an average period of five years after a single profile of glucose values pre- and post-meals, and demonstrated that hyperglycemia, measured two hours after lunch, but not pre-meal measures, was an independent risk factor for cardiovascular events (third vs. first tertile HRs 5.54, 95% CI 1.45 to 21.20 in women and 2.12, 95% CI 1.04 to 4.32 in men). In a later study, the same authors [9] followed 505 of these individuals with diabetes for about 14 years and assessed the relationship of postprandial glycemia with mortality. Hyperglycemia two hours after lunch was identified as an independent risk factor for cardiovascular mortality (individuals with postprandial glucose ≥ 180 mg/dL vs. <180 mg/dL, HR = 1.45, 95% CI: 1.06 to 1, 99) and overall mortality (HR = 1.85, 95% CI: 1.31 to 2.61).

Clinical trials aimed at minimizing postmeal glucose peaks have produced mixed findings in terms of the association of greater excursions with cardiovascular outcomes. Esposito et al. [21] randomized 175 patients to repaglinide or gyburide and subsequently followed them for 12 months, finding that post-prandial peaks were smaller (148 vs. 180 mg/dL), C-reactive protein and IL-6 lower, and the frequency of carotid artery intimal-media thickness regression greater (52% vs. 18%) in the repaglinide group, despite similar levels of HbA1_C_. On the other hand, lesser within day glucose variability was not associated with a reduction in cardiovascular events in a clinical trial investigating lispro (vs. NPH) insulin as a strategy to diminish postprandial glucose peaks [22]. However, the lispro intervention in this trial proved ineffective in significantly altering intraday variability, and the trial, stopped early for futility, and was of a relatively short duration.

Oxidative stress and inflammation have been suggested as potential mediators that could link postprandial hyperglycemia, in part through endothelial dysfunction [23-25], to diabetes complications [26-28] although the association of greater peaks with oxidative stress remains to be clearly established [29]. Individuals with diabetes tend to exhibit high levels of adhesion molecule markers of endothelial dysfunction – ICAM-1, VCAM-1 and E-selectin [30]. These markers, along with those of oxidative stress, increase after a test meal composed of fats (65 grams), glucose (75 grams), or the two together [31], this latter combination producing a greater effect than either of its parts in isolation.

Other studies have found a relationship between postprandial triglycerides and the complications of diabetes and/or risk factors for these complications. The triglyceride excursion after consumption of a test meal (729 kcal/m^2^; 65.2 grams of fat, 24.75 grams of carbohydrates) has been shown to be higher in individuals with diabetes; and among those with diabetes, to take longer to return to baseline in those who already have macrovascular complications [32]. Coutinho et al. [33] found that, in addition to an increase in triglyceride levels, consumption of a test meal rich in fat (682.6 kcal, 56.1 grams of fat, 34.4 grams of carbohydrates) was associated with an increase in blood leukocytes and a decrease in HDL-cholesterol. Teno et al. [11], in turn, found an association of postprandial triglyceride levels (following a standardized 9 kcal/kg meal) with the carotid intima-media thickness of patients with diabetes. Non-fasting triglycerides have been shown to predict cardiovascular events, especially in women [34]. We did not demonstrate any relationship of triglycerides (fasting, post-meal or excursion) with cardiovascular comorbidities, perhaps because our postprandial measure was two hours following meal consumption, too early to detect the triglyceride peak, which generally occurs four hours after consumption [35].

The recommended daily energy intake for our study participants (based on being sedentary with a median weight, height, and age) was 2310 kcal for men and 1755 kcal for women [36]. Nutritional recommendations generally allocate 20% of total energy intake to breakfast [37]. For our subjects, this would be 460 kcal to men and 351 kcal to women, approximately equal to the test meal used in our study – 454.8 kcal. This load, compared to others found in the literature, is small, but was adopted as it approximated what participants would be possibly taking in early morning if not being examined in our clinics, was easily standardized and was available at all investigation centers. Two of the test meal studies cited above used a load according to the body surface area which ranged from 700 kcal/m^2^ [31] to 730 kcal/m^2^ [32], resulting in 1211 to 1263 kcal load for a 1.73 m^2^ individual. As our participants median body surface area was 1.84 m^2^, this would translate to 1288 kcal to 1343 kcal. Others determined the load based on weight, and ranged from 9 kcal/kg [11] to 30 kcal/kg [38], equivalent to a 630 to 2100 kcal load for a 70 kg individual. Still others, which, like ours, used a standardized fixed test meal, also provided a higher number of calories, ranging from 500 [33] to 1480 kcal [39].

The relatively low energy intake in our study may thus have underestimated the true effect of usual postprandial glycemia in these mostly obese patients, especially if lunch and dinner meals are considered. Given that the caloric and fat content of meals usually taken by the overweight or obese are considerably greater than those we studied, it is likely that usual changes in blood glucose and triglycerides will be outside of the range we studied.

Another possible explanation for the small size of associations found with comorbidities was the source of saturated fat in the test meal of our study – dairy products. Higher consumption of saturated fats from dairy products, as assessed by a food frequency questionnaire, has been associated with lower risk of cardiovascular events (HR = 0.79, 95% CI: 0.68 to 0.92, for each increase of 5 grams/day) while consumption of saturated fat from meat is associated with an increased risk (HR = 1.26, 95% CI: 1.02-1.54, for each increase of 5 grams/day) [40]. Further, individuals already with prevalent comorbidity may have dampened their excursions through medication use, thus minimizing the associations we found.

It is also important to consider that both fasting and post-load glucose measures are extremely variable, not only between individuals, but also over time in a given individual [41]. As our study performed only a single measurement of plasma glucose and triglycerides, it cannot be expected to capture this biological variation, thus also potentially decreasing the size of associations seen [42,43].

In summary, a relatively small test meal, composed of foods common to breakfast with rapidly absorbed carbohydrate and saturated fat, produced variable postload excursions in a large sample of diabetic subjects, the magnitude of the glycemic excursions being correlated with factors suggesting insulin insufficiency. Though no associations with the presence of diabetes complications were found, small and at times statistically significant associations of greater excursions with comorbidity were seen in those with obesity. These findings suggest that postprandial excursions resulting from intake of rapidly absorbed carbohydrate and saturated fat of this magnitude, though perhaps without impact on diabetes complications in the non-obese, may be of importance in obese subjects with a lesser capacity for insulin secretion.
